# An Increasing Trend in the Prevalence of Polypharmacy in Sweden: A Nationwide Register-Based Study

**DOI:** 10.3389/fphar.2020.00326

**Published:** 2020-03-18

**Authors:** Naiqi Zhang, Jan Sundquist, Kristina Sundquist, Jianguang Ji

**Affiliations:** ^1^Center for Primary Health Care Research, Lund University/Region Skåne, Malmö, Sweden; ^2^Department of Family Medicine and Community Health, Department of Population Health Science and Policy, Icahn School of Medicine at Mount Sinai, New York, NY, United States; ^3^Center for Community-Based Healthcare Research and Education (CoHRE), Department of Functional Pathology, School of Medicine, Shimane University, Matsue, Japan

**Keywords:** polypharmacy, prevalence, temporal trend, national cohort, Sweden

## Abstract

**Aim:**

Polypharmacy is becoming a global health problem. The aims of this study were to evaluate the temporal trends in the prevalence of polypharmacy in Sweden and to explore polypharmacy disparities by age, gender, education, and immigration status.

**Methods:**

Polypharmacy and excessive polypharmacy were evaluated using data extracted from the Swedish Prescribed Drug Register between 2006 and 2014. Polypharmacy was defined as being exposed to five or more drugs and excessive polypharmacy was defined as being exposed to 10 or more drugs during 1 month respectively. Average annual percent change (AAPC) was calculated using Joinpoint Statistical Software.

**Results:**

The prevalence of polypharmacy increased from 16.9% in 2006 to 19.0% in 2014 with an AAPC of 1.3; the prevalence of excess polypharmacy increased from 3.8% in 2006 to 5.1% in 2014 with an AAPC of 3.4. The prevalence of polypharmacy and excessive polypharmacy increased dramatically with age and peaked up to 79.6% and 36.4% in individuals aged 90 and above respectively. Females and individuals with lower education level were associated with a higher rate of polypharmacy and excessive polypharmacy. Immigrants from Middle-Eastern countries had the highest rate of polypharmacy and excessive polypharmacy, whereas individuals from Western Europe countries had the lowest rate.

**Conclusion:**

The prevalence of polypharmacy has increased gradually in Sweden during the past decade. Individuals with older age, female sex, or lower education have a higher rate of polypharmacy and excessive polypharmacy. Immigrants from Middle-Eastern countries showed a higher rate of polypharmacy.

## Introduction

During recent decades, life expectancy for the world's population has increased dramatically, especially for the population in the developed countries (Mathers et al., 2015), when the leading causes of death shifted from infectious and acute diseases to non-communicable and chronic diseases (Collaborators, 2017). Along with population aging, the prevalence of multimorbidity as well as polypharmacy, i.e. individuals using different medications simultaneously, has increased gradually and become a global health problem (Lipska et al., 2016). Individuals with multimorbidity might benefit from concurrent use of different medications to improve the quality of life and longevity. However, multiple medications may lead to the increased risk of adverse drug reactions (Koh et al., 2005) and many unexpected negative effects caused by unknown drug interactions (Bushardt et al., 2008) and unnecessary health expenditure (Hovstadius and Petersson, 2013).

A previous study based on a national representative survey in the United States showed an alarming uptrend of polypharmacy from 1999 to 2012 (Kantor et al., 2015); this was consistent with another regional register-based study from the United Kingdom using data for the period between 1995 and 2010 (Guthrie et al., 2015). It is thus highly necessary to explore whether the uptrend of polypharmacy might be reliable based on data from nationwide registers and from countries with a national social welfare system, which could not be affected by selection and information bias. Previous studies of polypharmacy in Sweden mainly focused on elderly individuals (Johnell and Klarin, 2007; Haider et al., 2009; Wastesson et al., 2016; Morin et al., 2018; Wastesson et al., 2019), and studies included the whole population were outdated (Hovstadius et al., 2009; Hovstadius et al., 2010). Therefore, an updated assessment on the prevalence and temporal trend of multiple medications is highly needed. In addition, it is still largely unknown whether polypharmacy might be associated with common demographic factors, such as gender, education, and immigration status. In this study using Swedish data, we aimed to describe the temporal trend of polypharmacy using the entire Swedish population and to explore the associations of polypharmacy with gender, education, and immigration status.

## Materials and Methods

### Study Population

This study was approved by the Ethics Committee at Lund University, Sweden. The study population was the entire Swedish population who was alive on Jan 1st, 2006 or being born and migrated to Sweden between Jan 1st, 2006 and Dec 31st, 2014. We used the Swedish Prescribed Drug Register to evaluate multiple use of medications occurring among the entire Swedish population from 2006 to 2014. This register was created on 1st July 2005 and includes data on all prescribed drugs dispensed at pharmacies in Sweden (Ji et al., 2018; Huang et al., 2019). The rate of missing patient identity data is estimated to be lower than 0.3% (Wettermark et al., 2007).

In addition, we obtained sociodemographic characteristics, including age, sex, country of birth, and highest education level from Statistics Sweden's Total Population Register and Population Housing Census. The Swedish personal identification number, which is assigned upon birth in Sweden or, for immigrants, when registered in the Swedish population register, was used to link different registers and was then replaced with serial numbers to ensure people's integrity.

### Outcome Measurement

We defined polypharmacy as individuals exposed to five or more drugs during a calendar month, and excessive polypharmacy as individuals exposed to 10 or more drugs in a month (Hovstadius et al., 2009; Wastesson et al., 2019). As shown in **Supplementary Figure 1**, we calculated each individual's drug exposure during a specific calendar month using the following data: a) the date of drug dispensed; b) the defined daily doses per package; c) the number of dispensed packages. The duration of each dispensed drug was calculated by the defined daily doses per package multiplied by the number of dispensed packages. The number of different drugs used in each calendar month was retrieved according to seven-digit Anatomical Therapeutic Chemical Classification (ATC) codes.

## Statistical Analysis

To make the data comparable, the rates of polypharmacy and excessive polypharmacy were age-standardized using the Swedish population distribution in 2006 as the standard population. The study population was censored at the end of the study (Dec 31st, 2014), the date of death, or the date of emigration, whichever came first. Stratified results grouped by age (<60 years, 60–69 years, 70–79 years, 80–89 years, ≥90 years), sex, education levels, and country at birth were presented separately. Education level was classified into three groups, 0 to 9 years of schooling (elementary or compulsory education), 10 and 11 years of education (upper secondary education), and 12 years and above (higher education). Country at birth was grouped according to geographic areas: Sweden, Northern European country, Western European country, Eastern European country, Africa, Middle-Eastern country, and others. Prevalence rate ratios (PRRs) along with 95% confidence intervals (95% CIs) were calculated by dividing the prevalence rates in the study groups by that of the reference group.

We calculated the frequencies and ratios using Microsoft Excel (Office 365 MSO version 16.0). To assess the trend of polypharmacy and excessive polypharmacy during 2006–2014, we used joinpoint regression analysis which fitted a series of connected lines to the prevalence of polypharmacy and excessive polypharmacy. We joined straight-line segments at joinpoints, where the slope of prevalence trend significantly changed. If the trend of polypharmacy was a continuing straight-line without joint point, we presented it as missing. Average annual percent change (AAPC) was calculated as a geometrically weighted average of various annual percent change (APC) values from the regression analysis to describe the temporal trends of polypharmacy and excessive (Kim et al., 2000; Clegg et al., 2009). This analysis was conducted using Joinpoint Statistical Software (version 4.7.0.0).

## Results

**Table 1** shows the demographic and clinical characteristics of the study population during the study period from 2006 to 2014. The study population has increased from 9111430 in 2006 to 9747342 in 2017. Swedes accounted for 87.1% in 2006, and it was 83.5% in 2014. The top 10 drugs used in Sweden were phenoxymethylpenicillin, paracetamol, acetylsalicylic acid, diclofenac, simvastatin, omeprazole, metoprolol, furosemide, doxycycline, mucolytics.

**Table 1:** Demographic and clinical characteristics of Swedish population from 2006 to 2014.

	2006 (n,%)	2007 (n,%)	2008 (n,%)	2009 (n,%)	2010 (n,%)	2011 (n,%)	2012 (n,%)	2013 (n,%)	2014 (n,%)
Overall	9111430 (100)	9181434 (100)	9255323 (100)	9340105 (100)	9415487 (100)	9482760 (100)	9555806 (100)	9644811 (100)	9747342 (100)
Gender									
Male	4522476 (49.6)	4563049 (49.7)	4603116 (49.7)	4648716 (49.8)	4690246 (49.8)	4726808 (49.8)	4765887 (49.9)	4814338 (49.9)	4872228 (50.0)
Female	4588954 (50.4)	4618385 (50.3)	4652207 (50.3)	4691389 (50.2)	4725241 (50.2)	4755952 (50.2)	4789919 (50.1)	4830473 (50.1)	4875113 (50.0)
Age group									
< 60	6925232 (76.0)	6950399 (75.7)	6980437 (75.4)	7023907 (75.2)	7064110 (75.0)	7100679 (74.9)	7143970 (74.8)	7200729 (74.7)	7272492 (74.6)
60–69	1037351 (11.4)	1077136 (11.7)	1111646 (12.0)	1139190 (12.2)	1161824 (12.3)	1175885 (12.4)	1179493 (12.3)	1175241 (12.2)	1161103 (11.9)
70–79	658603 (7.2)	662943 (7.2)	670131 (7.2)	682627 (7.3)	692650 (7.4)	707978 (7.5)	734195 (7.7)	771126 (8.0)	814338 (8.4)
80–89	414648 (4.6)	414029 (4.5)	415137 (4.5)	414400 (4.4)	411160 (4.4)	408208 (4.3)	406916 (4.3)	404700 (4.2)	404412 (4.1)
90+	75596 (0.8)	76927 (0.8)	77972 (0.8)	79981 (0.9)	85743 (0.9)	90010 (0.9)	91232 (1.0)	93015 (1.0)	94996 (1.0)
Education, *year*									
0–9	3052708 (33.5)	3130921 (34.1)	3215295 (34.7)	3307538 (35.4)	3399741 (36.1)	3489885 (36.8)	3596265 (37.6)	3743101 (38.8)	3902423 (40.0)
10–11	3423742 (37.6)	3408622 (37.1)	3390714 (36.6)	3372882 (36.1)	3350355 (35.6)	3327298 (35.1)	3302523 (34.6)	3269693 (33.9)	3237560 (33.2)
12+	2634980 (28.9)	2641891 (28.8)	2649314 (28.6)	2659685 (28.5)	2665391 (28.3)	2665577 (28.1)	2657018 (27.8)	2632017 (27.3)	2607359 (26.8)
Birth country									
Sweden	7938009 (87.1)	7955099 (86.6)	7974718 (86.2)	8002682 (85.7)	8030609 (85.3)	8055524 (84.9)	8082598 (84.6)	8111318 (84.1)	8143852 (83.5)
Northern European country	273957 (3.0)	272629 (3.0)	269670 (2.9)	266519 (2.9)	263228 (2.8)	259748 (2.7)	256156 (2.7)	252054 (2.6)	248484 (2.5)
Western European country	112866 (1.2)	117131 (1.3)	122625 (1.3)	126691 (1.4)	128923 (1.4)	132849 (1.4)	137052 (1.4)	141795 (1.5)	145881 (1.5)
Eastern European country	242979 (2.7)	253645 (2.8)	263478 (2.8)	271210 (2.9)	277173 (2.9)	282846 (3.0)	288457 (3.0)	294510 (3.1)	302459 (3.1)
African country	59401 (0.7)	65032 (0.7)	71875 (0.8)	82686 (0.9)	93283 (1.0)	100056 (1.1)	108291 (1.1)	124477 (1.3)	133224 (1.4)
Middle-Eastern country	219967 (2.4)	237768 (2.6)	253500 (2.7)	267883 (2.9)	277860 (3.0)	287172 (3.0)	298629 (3.1)	317343 (3.3)	348217 (3.6)
Others	264251 (2.9)	280130 (3.1)	299457 (3.2)	322434 (3.5)	344411 (3.7)	364565 (3.8)	384623 (4.0)	403314 (4.2)	425225 (4.4)
Comorbidity									
Diabetes	240435 (2.6)	238353 (2.6)	237138 (2.6)	236554 (2.5)	237334 (2.5)	239225 (2.5)	240729 (2.5)	243127 (2.5)	248011 (2.5)
COPD	171402 (1.9)	169890 (1.9)	169002 (1.8)	168288 (1.8)	168037 (1.8)	167960 (1.8)	168288 (1.8)	168611 (1.7)	168987 (1.7)
CIHD	150720 (1.7)	154533 (1.7)	159092 (1.7)	163437 (1.7)	167926 (1.8)	172000 (1.8)	172637 (1.8)	180219 (1.9)	183622 (1.9)
Prescription drug use									
Phenoxymethylpenicillin	948147 (10.4)	991082 (10.8)	963254 (10.4)	889696 (9.5)	897428 (9.5)	905577 (9.5)	886665 (9.3)	789808 (8.2)	788097 (8.1)
Paracetamol	834350 (9.2)	857767 (9.3)	898881 (9.7)	942925 (10.1)	975973 (10.4)	999436 (10.5)	1047975 (11.0)	1096761 (11.4)	1154370 (11.8)
Acetylsalicylic acid	691013 (7.6)	708853 (7.7)	724567 (7.8)	732815 (7.8)	731154 (7.8)	721288 (7.6)	707108 (7.4)	682098 (7.1)	680786 (7.0)
Diclofenac	679818 (7.5)	640122 (7.0)	660245 (7.1)	656336 (7.0)	653810 (6.9)	643328 (6.8)	559870 (5.9)	446127 (4.6)	416384 (4.3)
Simvastatin	490985 (5.4)	553028 (6.0)	610378 (6.6)	679019 (7.3)	701853 (7.5)	701501 (7.4)	677366 (7.1)	634840 (6.6)	632482 (6.5)
Omeprazole	464277 (5.1)	517365 (5.6)	569537 (6.2)	613375 (6.6)	654976 (7.0)	704698 (7.4)	729480 (7.6)	734797 (7.6)	751624 (7.7)
Metoprolol	434802 (4.8)	464733 (5.1)	486946 (5.3)	502354 (5.4)	517579 (5.5)	533025 (5.6)	545623 (5.7)	554950 (5.8)	558831 (5.7)
Furosemide	389979 (4.3)	379300 (4.1)	373847 (4.0)	363981 (3.9)	355607 (3.8)	345988 (3.6)	338335 (3.5)	330374 (3.4)	329521 (3.4)
Doxycycline	370012 (4.1)	387912 (4.2)	345999 (3.7)	299605 (3.2)	301999 (3.2)	330838 (3.5)	310959 (3.3)	256672 (2.7)	248519 (2.5)
Mucolytics	358851 (3.9)	366866 (4.0)	343667 (3.7)	339617 (3.6)	405205 (4.3)	375345 (4.0)	333598 (3.5)	318780 (3.3)	324846 (3.3)

**Table 2** shows the prevalence of polypharmacy and excessive polypharmacy from 2006 to 2014. The rate of polypharmacy was 16.9% in 2006; it increased to 19.0% in 2014 with a significant AAPC of 1.3. The rate of excessive polypharmacy for the same period increased from 3.8% to 5.1% with a more remarkable AAPC of 3.4.

**Table 2:** Age-standardized rate of polypharmacy and excessive polypharmacy between 2006 and 2014.

	No. of individuals	Polypharmacy		Excessive polypharmacy	
		No.	%^a^	No.	%^a^
2006	9111430	1500452	16.9	333912	3.8
2007	9181434	1570231	17.5	356856	4.0
2008	9255323	1606057	17.7	371246	4.1
2009	9340105	1634487	17.8	380362	4.2
2010	9415487	1668156	18.0	386249	4.2
2011	9482760	1693447	18.1	390254	4.2
2012	9555806	1721687	18.2	397206	4.2
2013	9644811	1803681	18.8	440049	4.6
2014	9747342	1850219	19.0	497860	5.1

		AAPC	95% CI	AAPC	95% CI
Average Annual Percent Change (AAPC)		1.3	(1.0, 1.6)	3.4	(1.7, 5.1)

a Rates were adjusted according to the Swedish population distribution in 2006.

In 2014, the prevalence of polypharmacy and excessive polypharmacy was 79.6 and 36.4% respectively in age group 90 and above. Compared with individuals aged 0–59 years, the PRR of polypharmacy and excessive polypharmacy among individuals aged 90 and above was 11.24 (95%CI 11.13–11.36) and 31.21 (95%CI 30.78–31.66), respectively (**Table 3**). The rate of polypharmacy and excessive polypharmacy in females was continuously higher than males during the study period, and the PRRs (95% CIs) were 1.20 (95% CIs 1.20–1.20) and 1.26 (95% CIs 1.25–1.26), respectively. The rate of polypharmacy and excessive polypharmacy was lowest among individuals with the highest education, and the PRRs (95% CIs) were 0.77 (0.77, 0.77) for polypharmacy and 0.66 (0.65, 0.66) for excessive polypharmacy compared with individuals with the lowest education. Immigrants from Middle-Eastern countries had the highest rates of polypharmacy and excessive polypharmacy, and the PRRs were 1.21 (95%CI 1.20–1.22) and 1.63 (95%CI 1.61–1.65) respectively compared with native Swedes. Immigrants from Western European countries had the lowest rates; a PRR of 0.80 (95%CI 0.79–0.81) for polypharmacy and 0.83 (95%CI 0.81–0.85) for excessive polypharmacy (**Table 3**).

**Table 3:** Prevalence rate ratio of polypharmacy and excessive polypharmacy in 2014.

	Polypharmacy			Excessive polypharmacy		
	Rate^a^	PRR	95% CI	Rate^a^	PRR	95% CI
Gender						
Male	17.3	1		4.5	1	
Female	20.7	1.20	1.20–1.20	5.7	1.26	1.25–1.26
Age group						
< 60	8.5	1		1.4	1	
60–69	35.9	4.52	4.50–4.54	9.2	6.99	6.93–7.05
70–79	54.8	7.61	7.57–7.64	17.0	14.32	14.20–14.44
80–89	73.0	8.01	7.96–8.05	29.0	19.32	19.14–19.49
90+	79.6	11.24	11.13–11.36	36.4	31.21	30.78–31.66
Education, year						
0–9	21.3	1		6.2	1	
10–11	19.2	0.90	0.90–0.90	5.2	0.82	0.82–0.83
12+	16.5	0.77	0.77–0.77	4.1	0.66	0.65–0.66
Birth country						
Sweden	18.9	1		5.0	1	
Northern European country	20.1	1.06	1.05–1.07	6.0	1.19	1.17–1.21
Western European country	15.2	0.80	0.79–0.81	4.1	0.83	0.81–0.85
Eastern European country	18.8	0.99	0.99–1.00	6.0	1.21	1.19–1.22
African country	16.8	0.89	0.87–0.90	4.5	0.90	0.87–0.92
Middle-Eastern country	23.0	1.21	1.20–1.22	8.1	1.63	1.61–1.65
Others	16.8	0.89	0.88–0.89	5.0	1.00	0.99–1.01

a Rates were adjusted according to the Swedish population distribution in 2006.

**Figure 1:**
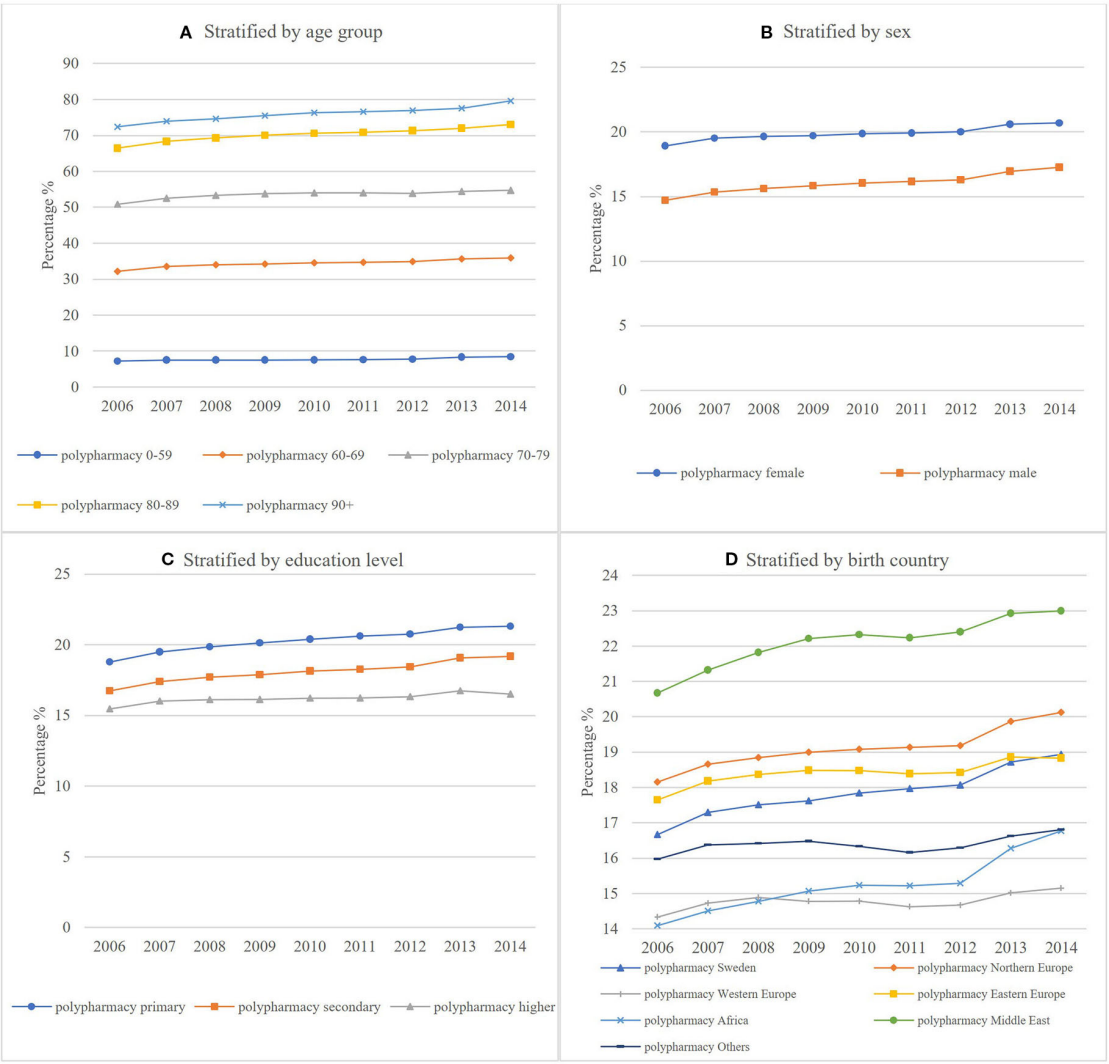
Age-standardized rate of polypharmacy stratified by **(A)** age group **(B)** sex **(C)** education level **(D)** birth country.

**Figure 2:**
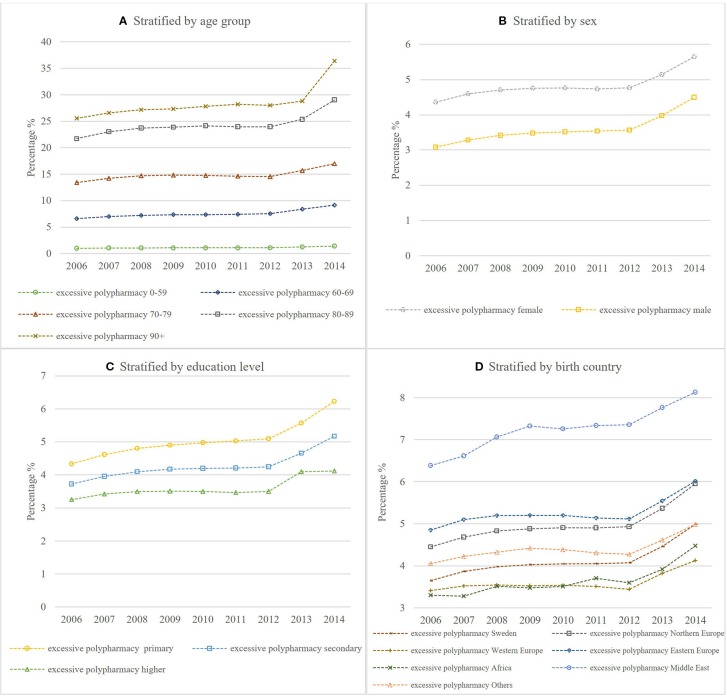
Age-standardized rate of excessive polypharmacy stratified by **(A)** age group **(B)** sex **(C)** education level **(D)** birth country.

**Figures 1** and **2** show the temporal trend of polypharmacy and excessive polypharmacy stratified by age group, sex, education level, and birth country respectively. The rate of polypharmacy and excessive polypharmacy increased significantly in all age groups during the study period from 2006 to 2014 (**Tables 4** and **5**).

**Table 4:** Results of joinpoint trend analysis for polypharmacy.

	Average Annual Percent Change (AAPC) (95% CI)	Trend 1		Trend 2	
		Duration	Annual Percent Change (APC) (95% CI)	Duration	Annual Percent Change (APC) (95% CI)
Overall	1.3 (1.0, 1.6)	–	–	–	–
Gender					
Male	1.8 (1.4, 2.1)	–	–	–	–
Female	1.0 (0.7, 1.3)	–	–	–	–
Age group					
0–59	2.0 (1.1, 2.9)	2006–2012	1.1 (0.3, 1.8)	2012–2014	4.9 (0.5, 9.4)
60–69	1.3 (0.9, 1.7)	2006–2008	2.5 (0.5, 4.5)	2008–2014	0.8 (0.5, 1.2)
70–79	0.9 (0.6, 1.2)	2006–2008	2.5 (0.9, 4.0)	2008–2014	0.3 (0.1, 0.6)
80–89	1.1 (0.8, 1.4)	2006–2008	2.1 (0.6, 3.6)	2008–2014	0.8 (0.5, 1.0)
90+	1.0 (0.8, 1.2)	–	–	–	–
Education level, year					
0–9	1.6 (1.2, 1.9)	2006–2008	2.8 (1.1, 4.6)	2008–2014	1.2 (0.9, 1.5)
10–11	1.5 (1.2, 1.8)	–	–	–	–
12+	0.7 (0.4, 1.1)	–	–	–	–
Birth country					
Sweden	1.4 (1.1, 1.7)	–	–	–	–
Northern European country	1.1 (0.7, 1.4)	–	–	–	–
Western European country	0.4 (0, 0.8)	–	–	–	–
Eastern European country	0.6 (0.2,0.9)	–	–	–	–
African country	2.0 (1.2, 2.8)	2006–2012	1.3 (0.6, 1.9)	2012–2014	4.3 (0.3, 8.4)
Middle-Eastern country	1.3 (0.6, 1.9)	2006–2008	2.8 (−0.4, 6.1)	2008–2014	0.8 (0.2, 1.3)
Others	0.4 (0, 0.7)	–	–	–	–

**Table 5:** Results of joinpoint trend analysis for excessive polypharmacy.

	Average Annual Percent Change (AAPC) (95% CI)	Trend 1		Trend 2	
		Duration	Annual Percent Change (APC) (95% CI)	Duration	Annual Percent Change (APC) (95% CI)
Overall	3.4 (1.7, 5.1)	2006–2012	1.5 (0.1, 3.0)	2012–2014	9.2 (0.3, 18.8)
Gender					
Male	4.3 (2.5, 6.2)	2006–2012	2.2 (0.6, 3.7)	2012–2014	11.0 (1.6, 21.4)
Female	2.8 (1.2, 4.5)	2006–2012	1.2 (−0.2, 2.6)	2012–2014	8.0 (−0.6, 17.2)
Age group					
0–59	4.4 (3.3, 5.5)	2006–2012	1.7 (0.9, 2.6)	2012–2014	12.7 (7.0, 18.7)
60–69	3.8 (2.1, 5.5)	2006–2012	1.9 (0.5, 3.4)	2012–2014	9.6 (0.7, 3.0)
70–79	2.0 (0.9, 3.2)	–	–	–	–
80–89	3.0 (1.2, 4.8)	2006–2012	1.2 (−0.3, 2.8)	2012–2014	8.3 (−0.9, 18.4)
90+	3.7 (2.8, 4.5)	2006–2012	1.1 (0.4, 1.8)	2012–2014	11.8 (7.4, 16.5)
Education level, year					
0–9	4.1 (2.5, 5.8)	2006–2012	2.4 (1.1, 3.8)	2012–2014	9.4 (1.0, 18.5)
10–11	3.7 (1.9, 5.5)	2006–2012	1.9 (0.4, 3.4)	2012–2014	9.1 (−0.1, 19.2)
12+	2.5 (1.0, 4.0)	–	–	–	–
Birth country					
Sweden	3.5 (1.8, 5.2)	2006–2012	1.5 (0.1, 3.0)	2012–2014	9.6 (0.7, 19.3)
Northern European country	3.2 (1.7, 4.8)	2006–2012	1.4 (0.1, 2.8)	2012–2014	8.8 (0.7, 17.5)
Western European country	2.2 (1.1, 3.3)	2006–2012	0.1 (−0.8, 1.0)	2012–2014	8.7 (3.0, 14.7)
Eastern European country	2.2 (0.5, 3.9)	2006–2012	0.6 (−0.9, 2.0)	2012–2014	7.3 (−1.4,16.7)
African country	3.7 (2.0, 5.4)	2006–2012	1.7 (0.3, 3.1)	2012–2014	10.0 (1.4, 19.3)
Middle-East country	2.6 (1.8, 3.4)	–	–	–	–
Others	1.8 (0.6, 2.9)	–	–	–	–

## Discussion

In this nationwide register-based study, a comprehensive assessment of the temporal trend in the prevalence of polypharmacy and excessive polypharmacy found that the prevalence increased gradually from 2006 to 2014. The increase was independent of gender, age, education, and immigration status, which suggests that the increased prevalence of the changes concerning polypharmacy might reflect the development of new clinical guidelines, the improved health needs of the general population, as well as the enhanced understanding of the importance of disease prevention. In addition, we found that females, individuals with a lower education, and immigrants from Middle-Eastern countries to Sweden had a higher prevalence of polypharmacy.

It should be noted that the measurement and definition of polypharmacy varied from different studies. However, our finding of an increased prevalence of polypharmacy is consistent with studies from other developed countries (Fincke et al., 2005; Gillette et al., 2015; Masnoon et al., 2017). In the National Health and Nutrition Examination Survey from the United States, the percentage of adults aged 20 and older that had polypharmacy increased remarkably from 8.2% in 1999–2000 to 15.0% in 2011–2012 and they defined polypharmacy as using five or more prescription drugs 30 days prior to the survey interview (Kantor et al., 2015). A population database analysis from the United Kingdom showed that the proportion of adults that reported polypharmacy (dispensed five or more drugs) almost doubled from 11.2% in 1995 to 20.8% in 2010, and the proportion of excessive polypharmacy (dispensed 10 or more drugs) tripled from 1.7 to 5.8% (Guthrie et al., 2015) during this same period. A Swedish nationwide study based on data retrieved from the Swedish Prescribed Drug Register reported that the rate of individuals who were prescribed five or more drugs within a 3-month period increased from 10.2 to 11.1%, and the rate of individuals who were prescribed 10 or more drugs within a 3-month period increased from 2.1 to 2.4% (Hovstadius et al., 2010). With the development of medical care, a growing number of chronic diseases can be treated thus resulting in an increasing number of people living with multimorbidity and taking multiple medications at the same time to treat each condition. Besides, the increased application of primary and secondary preventative strategies also improved multiple medication use (Mantel-Teeuwisse et al., 2001; Gorard, 2006). Although many interventions have been implemented to reduce polypharmacy, the results have been unsatisfactory (Kalisch et al., 2011; Tamura et al., 2011; Radcliff et al., 2015; Nguyen and Spinelli, 2016; Antimisiaris and Cutler, 2017).

We found that polypharmacy and excessive polypharmacy is becoming more common not only in older people but also in younger individuals. The prevalence of polypharmacy and excessive polypharmacy increased dramatically with age, peaking up to 79.6% and 36.4% in individuals aged 90 and above (Morin et al., 2018). The elderly are more likely to live with multiple chronic conditions thus leading to multiple transitions of health care and polypharmacy. Therefore, the high prevalence of multiple medication use might be associated with the relatively large number of individuals living with multimorbidity rather than due to ageing (Linjakumpu et al., 2002; Hajjar et al., 2007).

Our findings suggested that a higher prevalence of polypharmacy and excessive polypharmacy exists among females, which is consistent with previous studies (Bjerrum et al., 1998; Venturini et al., 2011; Costa et al., 2017; Midao et al., 2018). The possible explanation might be as follows: First, females have a life expectancy advantage over males and they have to live with chronic diseases for a longer period of time (Kontis et al., 2017). Second, females may pay more attention to their health conditions and consequently are more likely to report signs and symptoms to health professionals, which often results in multiple medication use (Santalucia et al., 2015). In addition, women are more likely to participate in preventive health care and are more likely to be prescribed for primary and secondary prevention (Granger et al., 2009).

Individuals with a higher education level were less likely to have polypharmacy and excessive polypharmacy. Some published studies in Sweden reported similar results (Haider et al., 2007; Haider et al., 2009). Furthermore, studies from Malaysia (Ong et al., 2018) and Pakistan (Sarwar et al., 2018) also suggested that older patients with low level education were significantly associated with a higher rate of polypharmacy. The potential explanation might be that patients with a lower level of education are less aware of their basic health needs and expect clinicians to prescribe a greater number of medications (Abdulrahman, 2003). However, it could also be related to a better health in individuals with higher education and a lower need of pharmacological therapy.

We found that the age-standardized rate of polypharmacy and excessive polypharmacy were highest among immigrants from Middle-Eastern countries, and lowest among immigrants from Western European countries. Reports from Israel (Beloosesky et al., 2013) and Turkey (Bahat et al., 2014) observed a relatively high prevalence of polypharmacy, with a rate of 42.6% among Israelis aged 65 and over and 47.6% among Turks aged 60 and over, while the rate was much lower in the United Kingdom (8.7% for age group 60–69, 17.1% for age group 70–79, 24.0% for age group 80+) (Guthrie et al., 2015) and France (Herr et al., 2017) (21.1% for individuals aged 65 and above).

Polypharmacy is usually associated with numerous negative clinical consequences, especially in the elderly. Previous research has clearly established that the concurrent use of multiple medications may result in the increasing risk of adverse drug event both in outpatients (Bourgeois et al., 2010) and hospitalized patients (Marcum et al., 2012). Besides, individuals with polypharmacy are predisposed to drug-drug interactions, and the risk increased with the number of medications (Mallet et al., 2007). The rate of non-adherence with drugs (Vik et al., 2004) as well as inappropriate prescribing (Bao et al., 2012) was also associated with polypharmacy, which may lead to consequently subsequent adverse health outcomes such as potential disease progression, treatment failure, and hospitalization. In addition, the unnecessary health expenditure caused by polypharmacy also imposes a huge financial burden on the healthcare system (Hovstadius and Petersson, 2013). Considering the increased risk of health problems caused by polypharmacy, clinicians should balance the benefits and potential harms when prescribing drugs against multimorbidity. The APC of polypharmacy and excessive polypharmacy was higher in 2012–2014 compared with that in 2006–2011. One possible reason was that following the Syrian Civil War in 2011 many Syrians arrived Sweden as asylum seekers. According to Statistics Sweden, 116,384 citizens of Syria (70,060 men, 46,324 women) were residing in Sweden in 2016. These immigrants from Syria might experience posttraumatic stress disorder and had a higher prevalence of polypharmacy or excessive polypharmacy. However, the joinpoint analysis was data driven may be due to spurious reasons although we did our best to identify potential drivers of the change in excessive polypharmacy. We have provided a comprehensive picture of multiple use of prescribed drugs in the entire Swedish population using individual-based exposure of dispensed drugs. The assessments of monthly exposure to polypharmacy provide more accurate information in multiple medication use status than earlier studies only using the data on prescribed date (Johnell and Klarin, 2007; Haider et al., 2009; Hovstadius et al., 2009; Hovstadius et al., 2010). However, there are several limitations that warrant consideration. First, the Swedish Prescribed Drug Register does not include information on over the counter medications and drugs used in hospitals, which may lead to an underestimation of the individual burden of polypharmacy. Besides, we only obtained the information about the drugs being dispensed, but we could not know whether the drugs were actually consumed or the exact date when patients start to take the medications; this may result in misclassification of polypharmacy.

In conclusion, a significant increase of polypharmacy and excessive polypharmacy was found among the Swedish population during the past decade. Individuals with older age, female sex, and lower education may have a higher rate of polypharmacy and excessive polypharmacy. The rates were highest among immigrants from Middle-Eastern countries, and lowest among immigrants from Western European countries. The causes behind this polypharmacy and the sociodemographic disparities need to be further examined.

## Data Availability Statement

The datasets generated for this study are available on request to the corresponding author.

## Ethics Statement

The studies involving human participants were reviewed and approved by the Ethics Committee at Lund University. Written informed consent to participate in this study was provided by the participants' legal guardian/next of kin.

## Author Contributions

NZ, JS, KS, and JJ were responsible for the study concept and design. JS, KS, and JJ obtained funding. KS and JS acquired the data. NZ did the statistical analysis and drafted the manuscript, and all authors revised it for important intellectual content. The authors confirm that the Principal Investigator for this paper is JJ.

## Funding

This work was supported by grants awarded to JJ by the Swedish Research Council (2016-02373), Cancerfonden (CAN2017/340), and Crafoordska stiftelsen, to KS by the Swedish Research Council, and to JS by the Swedish Research Council as well as by ALF funding from Region Skåne awarded to JS, KS, and JJ.

## Conflict of Interest

The authors declare that the research was conducted in the absence of any commercial or financial relationships that could be construed as a potential conflict of interest.
