# Supplementary figures and images for: An Increasing Trend in the Prevalence of Polypharmacy in Sweden: A Nationwide Register-Based Study
Source: Front Pharmacol. 2020 Mar 18;11:326. doi: 10.3389/fphar.2020.00326 (PMC7103636; doi:10.3389/fphar.2020.00326)

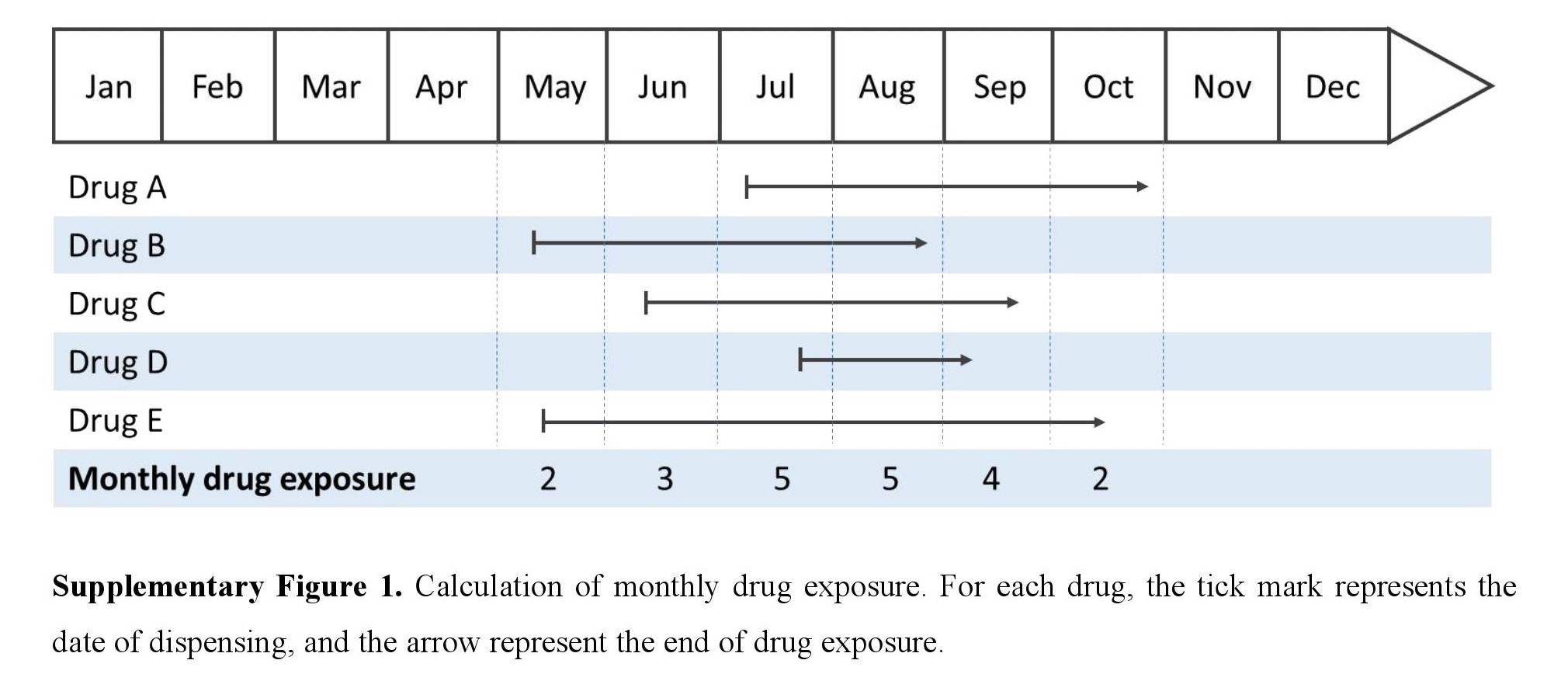

Supplement: Supplementary file 1 [file Image_1.jpeg]
